# Cryptozoospermia: Should we use ejaculated sperm or surgically retrieved sperm for assisted reproductive technology?

**DOI:** 10.1002/rmb2.12546

**Published:** 2023-10-26

**Authors:** Hastuki Hibi, Mikiko Tokoro, Megumi Sonohara, Kazuho Ihara, Noritaka Fukunaga, Yoshimasa Asada

**Affiliations:** ^1^ Department of Urology Kyoritsu General Hospital Nagoya Japan; ^2^ Asada Ladies Clinic Nagoya Japan; ^3^ Asada Institute for Reproductive Medicine Nagoya Japan

**Keywords:** assisted reproductive technology, cryptozoospermia, ejaculated sperm, intracytoplasmic sperm injection, sperm retrieval surgery

## Abstract

**Purpose:**

In cryptozoospermic subjects, it may often may be difficult to secure motile sperm for assisted reproductive technology (ART). We examined the results of ART with frozen thawed ejaculated sperm in cryptozoospermic subjects and evaluated whether sperm retrieval surgery is necessary for such patients in our clinic.

**Methods:**

Between 2013 and 2021, we evaluated 197 cryptozoospermic patients. Age, endocrine panel at the time of the initial semen analysis, and anti‐müllerian hormone levels at the time of the spouse's first egg retrieval were examined. Cryopreservation of ejaculated motile sperm collected essentially weekly over a 3‐month period was carried out. ART data recorded was the number of egg retrieval cycles, normal fertilization rate, and clinical pregnancy rate.

**Results:**

ART using frozen sperm as well as sperm ejaculated on the day of egg retrieval was possible in all cases. The normal fertilization rate was 70.4%, the clinical pregnancy rate per embryo transferred was achieved in 31.5% (870 cycles), and the live birth rate per case was 73.8%.

**Conclusions:**

Intracytoplasmic sperm injection (ICSI) was possible without sperm retrieval surgery in cryptozoospermia, resulting in 73.8% of live births per patient. Sperm identification, sperm processing, and ICSI technique are especially important in cryptozoospermia. Sperm retrieval surgery can be avoided in cryptozoospermic patients.

## 1 INTRODUCTION

The strategy for azoospermic patients to obtain sperm is undoubtedly sperm retrieval surgery such as microscopic epididymal sperm aspiration and testicular sperm extraction (TESE). If there is a treatable disease such as varicocele, treatment should be prioritized and improvement of semen findings should be expected. However, there is no established treatment in the cases of idiopathic oligoasthenozoospermia. Cryptozoospermia is defined as a condition in which spermatozoa are absent from fresh preparations but observed after repeated examination in a centrifuged pellet. In patients with a limited number of motile sperms, intracytoplasmic sperm injection (ICSI) is the treatment of choice to achieve pregnancy. However, there is an opinion that the DNA damage of ejaculated sperm causes poor assisted reproductive technology (ART) results. To investigate whether poor ART results are due to sperm, we evaluated the ART results with the use of ejaculated sperm from cryptozoospermic patients in our institute.

## 2 MATERIALS AND METHODS

### 2.1 Study design, size, and duration

In total, 199 patients diagnosed with cryptozoospermia at the Asada Ladies Clinic over a 9‐year period between 2013 and 2021 were evaluated. Subjects who demonstrated adequate sperm parameters at the time of ART were excluded, and ethical informed consent was required to be included in the study.

### 2.2 Patients and setting

Diagnosis of cryptozoospermia was according to the 5th edition of the WHO laboratory manual^1^ as a condition in which spermatozoa are absent from fresh preparations but observed after repeated examination in a centrifuged pellet. Ejaculates were collected for cryopreservation attempts, essentially weekly for 3 months, until there was sufficient sperm to obtain several samples. ART results were compared with 35 patients with non‐obstructive azoospermia (NOA) who underwent micro‐TESE and had motile sperm recovered during the same study period.

We examined the patient's age at the time of initial semen analysis, endocrine panel, spouse's age at the initial egg retrieval, and anti‐müllerian hormone (AMH). The results of ART were evaluated according to number of embryo transfers, embryo transfer cycle, normal fertilization rate, clinical pregnancy rate, and clinical pregnancy rate per case.

### 2.3 Sperm counting, freezing, and thawing

Initially, the collected sperm were counted using a microscope. For non‐frozen sperm, they were utilized for insemination immediately after the counting process. To freeze the sperm, the following steps were followed: The sperm were washed multiple times with HEPES medium (Sperm Washing Medium, SW012, Nakamedical Inc.) and then centrifuged at 300 *g* for 10 min. After the centrifugation, the supernatant was removed, leaving behind the pelleted sperm. The pelleted sperm were then suspended in a sperm‐freezing solution (Freezing Medium, 90 128, FUJIFILM IrvineScientific, Inc.). Special sperm freezing straws (Sperm Freeze Straw 0.5 mL, NFA101, Nakamedical Inc.) were utilized for the freezing process. The straws were filled with PBS, air, sperm solution, air, and PBS in that precise order. To seal the ends of the straws, straw powder (Straw Powder, FA349, Nakamedical Inc.) was applied. After a 10‐min wait, the straws containing the sperm were cryopreserved in liquid nitrogen. Thawing of the sperm was conducted using the following steps: The straws with the sperm solution were taken out of the liquid nitrogen and left at room temperature for 30 s. Afterward, they were placed in hot water at 30–35°C for 1 min. Using scissors, the straws were then cut, and the sperm were collected in 15‐mL tubes. HEPES medium was added to the sperm solution, lightly mixed, and then centrifuged at 300 *g* for 10 min. The supernatant was removed after the centrifugation, leaving the sperm in pellet form. The sperm pellets were resuspended in pentoxifylline solution adjusted to 3.6 mM in HEPES medium and subsequently used for insemination. There is no difference between testicular sperm and cryptozoospermia sperm in methods of sperm counting, freezing, and thawing.

### 2.4 Protocol for controlled ovarian stimulation

Controlled ovarian stimulation (COS) was conducted using either Human Menopausal Gonadotropin (Ferring Pharmaceuticals or ASKA Pharmaceutical) or recombinant FSH (Gonal‐f®; Merck Serono).^2^ For the long protocol, nasal buserelin at a dosage of 600 μg/day (Buserecur®; Fuji Pharma) was administered on the prestimulation cycle day 21. The short protocol was also used and involved nasal buserelin at a dosage of 600 μg/day on the stimulation cycle day 3. In both protocols, a GnRH agonist was administered daily until the day of hCG injection. In the GnRH antagonist protocol, a GnRH antagonist (ganirelix [Ganirest®, MSD] or cetrorelix [Cetrotide®, Merck Serono]) was administered alternate days (in principle) when the follicles reached a diameter of 14–16 mm or if a premature LH surge was suspected based on blood LH levels. Following this, final oocyte maturation was induced either with a GnRH agonist or hCG (3000–5000 IU). After 34–36 h from hCG injection, mature oocytes were collected trans‐vaginally, depending on factors such as follicular diameter, serum E2 and AMH levels, and the patient's age. The retrieved oocytes were then fertilized by ICSI. Fresh embryo transfer was carried out if the following criteria were met: (a) the serum E2 level on the day of hCG injection was ≤6000 pg/mL, (b) the serum P4 level on the day of hCG injection was ≤1.5 ng/mL, and (c) there were no signs of Ovarian Hyperstimulation Syndrome observed after oocyte retrieval.

### 2.5 Embryo culture and cryopreservation

During fresh embryo transfers, each embryo was cultured individually from days 3 to 5. For freeze‐thawed embryo transfers, embryos were frozen either at the pronuclear stage or as blastocysts, employing two distinct methods: slow freezing and vitrification.

The slow freezing process was performed using specialized equipment, namely the Embryo Freezing Pack and Embryo Thawing Pack (Origio). The vitrification method utilized the Vitrification Kits VT101 and VT102 (Kitazato BioPharma) to ensure successful cryopreservation of embryos.

### 2.6 Embryo transfer

In frozen–thawed embryo transfers, artificial hormone replacement cycles were employed, following a specific endometrial preparation protocol using a combination of transdermal estradiol (Estrana®, Hisamitsu) and chlormadinone acetate (Lutoral®, FujiPharma).^3^ The treatment with Estradiol began on either the second or third day of the artificial hormone replacement cycle. The endometrial thickness was then measured between days 9 and 11 of the cycle. If the endometrium thickness measured ≥7 mm, the frozen–thawed embryo transfer was scheduled. On day 15 of the cycle, chlormadinone acetate treatment commenced with a daily dose of 6 mg. Cleavage‐stage embryos were transferred on day 3, considering the starting day of chlormadinone acetate treatment as day 0. Alternatively, blastocyst‐stage embryos were transferred on day 6, also considering the starting day of chlormadinone acetate treatment as day 0. In cases of fresh embryo transfers, if pregnancy occurred, transdermal estradiol was administered at a dose of 2.16 mg every 2 days, along with chlormadinone acetate at a dose of 12 mg/day. Both for fresh and frozen–thawed embryo transfers, transdermal estradiol at a dose of 2.16 mg every 2 days and chlormadinone acetate at a dose of 6 mg/day were continued until 9 weeks of gestation. To confirm pregnancy, urinary hCG measurements and transvaginal ultrasound scans were performed. A urinary hCG level of ≥50 IU/mL on day 14 after embryo transfer was considered a positive pregnancy test and the presence of an intrauterine gestational sac was verified 1 week later through a transvaginal ultrasound scan.

### 2.7 Institutional review board

The protocol for this research project, including its use of human subjects, was approved by a suitably constituted Asada Ladies Clinic Ethical Committee. Our approval number is 2020‐14, and the date of approval is September 30, 2020.

## 3 RESULTS

Median patient age and spouse were 37 (interquartile range: 34, 43), 36 (31, 39) in cryptozoospermia cases, and whereas were 36 (31, 43), 33 (31, 38) in NOA cases who received micro‐TESE. The median AMH of the spouse was 3.57 ng/mL (0.00–15.10) in cryptozoospermia cases and 3.49 ng/mL (0.24, 14.59) in TESE. The median testicular volume (right/left) was 12 (10, 16)/12 (8, 14) mL in cryptozoospermia cases, and 8 (5, 11)/6 (5, 10) mL in NOA. The median endocrine panel of luteinizing hormone (LH), follicle‐stimulating hormone (FSH), testosterone was 6.5 mIU/mL (4.7, 9.4), 8.0 mIU/mL (5, 17), 4.26 ng/mL (3.28, 5.35) in cryptozoospermia cases, and 10.6 mIU/mL (5.8, 15.9), 22.0 mIU/mL (13, 33), 3.45 ng/mL (2.59, 5.06) in TESE, respectively. Details of patient characteristics are shown in Table 1. Between cryptozoospermia and TESE subjects, there was no difference in age, testosterone levels, spouse age, and AMH levels, however, LH and FSH were significantly higher and decreased testicular volume in TESE subjects.

**TABLE 1:** Patient characteristics.

	Cryptozoospermia (*n* = 199)	TESE (*n* = 35)	*p*‐value^a^
Age (years)	37 (34, 43)	36 (31, 43)	0.15
Souse age (years)	35 (31, 39)	33 (31, 38)	0.264
Spouse AMH (ng/mL)	3.33 (1.29, 3.82)	3.01 (1.11, 4.46)	0.607
Testicular volume right/left (mL)	12 (10, 16)/12 (8, 14)	8 (5, 11)/6 (5, 10)	0.001/0.002
LH (mIU/mL)	6.5 (4.7, 9.4)	10.6 (5.8, 15.9)	0.002
FSH (mIU/mL)	8.3 (5.1, 16.7)	22.4 (13.1, 33.2)	<0.001
Testosterone (ng/mL)	4.26 (3.28, 5.35)	3.45 (2.59, 5.06)	0.052

*Note*: Each variable is expressed as a median (IQR).

Abbreviations: AMH, anti‐müllerian hormone; FSH, follicle‐stimulating hormone; LH, luteinizing hormone.

^a^ Mann–Whitney *U* test.

We provide detailed information about the sperm used for insemination in Table 2. The same sperm freezing and thawing methods were employed for both the Cryptozoospermia and TESE groups. In the Cryptozoospermia group, a total of 4350 straws were used to preserve sperm in 195 cases, but only 167 straws were thawed and used for insemination. Of the 4545 oocytes inseminated by ICSI, 3535 (77.8%) were inseminated using fresh ejaculated sperm. The remaining 1010 oocytes (22.2%) were inseminated using thawed sperm. In the TESE group, all inseminations were performed using thawed sperm.

**TABLE 2:** Sperm parameters summary.

	Cryptozoospermia (*n* = 197)^a^	TESE (*n* = 35)^a^
Total number of frozen sperm straws	4350	682
Median number of frozen sperm straws	17 (10, 30)	20 (8, 26)
Total number of thawed sperm straws	167	68
Median number of thawed sperm straws	0 (0, 1)	2 (1, 2)
Number of total sperm used ICSI	4545	606
Fresh,*n* (%)	3535 (77.8)	0 (0)
Frozen–thawed, *n* (%)	1010 (22.2)	606 (100)
Median number of sperm used ICSI	20 (12, 29)	18 (11, 22.5)

	Fresh	Frozen–thawed	Frozen–thawed
Motility of Sperm used for insemination, *n* (%)			
Motile sperm	3411 (96.5)	890 (88.1)	480 (79.2)
Weakly motile sperm	18 (0.5)	74 (7.3)	96 (15.8)
Non‐motile sperm	106 (3.0)	46 (4.6)	30 (5.0)
Sperm morphology, *n* (%)			
Normal	3498 (99.0)	920 (91.1)	510 (84.2)
Abnormal	37 (1.0)	90 (8.9)	96 (15.8)

Abbreviation: ICSI, intracytoplasmic sperm injection.

^a^ Median (IQR); *n* (%).

Among the sperm used for insemination, 88.1% in the Cryptozoospermia group and 79.2% in the TESE group exhibited motility, while the proportion of morphologically normal sperm was 91.1% in the Cryptozoospermia group and 84.2% in the TESE group.

All subjects were scheduled to receive ART using frozen thawed ejaculated sperm or ejaculated sperm on the day of egg retrieval. Details of ART results are shown in Table 3. Micro‐TESE was required only in one case where there was a desire for a second child due to lack of cryopreserved sperm. Although this subject received micro‐TESE, no sperm were recovered. In the comparison of ART results between cryptozoospermia and TESE, the number of mature oocytes and the number of two‐pronuclear embryos were lower in cryptozoospermia cases, but no difference was observed in the normal fertilization rate (70.4% vs. 72.1%, *p* = 0.4182). Similarly, cryptozoospermia cases had lower clinical pregnancy and birth rates per embryo‐transfer cycle, but there was no difference in live birth rates per patient (73.8% vs. 87.5%, *p* = 0.118).

**TABLE 3:** Results of ART.

Characteristic	Cryptozoospermia (*n* = 197)^a^	TESE (*n* = 35)^a^	*p*‐value
Female age (years)	35 (31, 39)	33 (31, 38)	0.264^b^
Number of IVF cases performed	197	35	—
Number of OPU cycles	502	59	—
Total number of mature oocytes	4663	606	—
Median number of mature oocytes	4 (2, 13)	8 (4, 16)	0.00148^b^
Total number of two‐pronuclear embryos	3284	437	—
Median number of two‐pronuclear embryos	3 (1, 10)	6 (4, 10)	0.00112^b^
Two‐pronuclear fertilization rate, *n* (%)	3284/4663 (70.4)	437/606 (72.1)	0.4182^b^
Embryo transfer cycles	871	117	—
Cleavage‐stage embryo transfer, *n* (%)	409 (47.0)	52 (44.4)	—
Blastocyst‐stage embryo transfer, *n* (%)	462 (53.0)	65 (55.6)	—
Implantation rate/ET cycle^d^, *n* (%)	312/870 (35.9)	58/117 (49.6)	0.005528^c^
Clinical pregnancy rate/ET cycle^d^, *n* (%)	274/870 (31.5)	51/117 (43.6)	0.01211^c^
Live birth rate/ET cycle^d^, *n* (%)	172/841 (20.5)	35/112 (31.3)	0.01308^c^
Live birth rat /patient^e^, *n* (%)	135/183 (73.8)	28/32 (87.5)	0.118^c^

Abbreviations: ET, embryo transfer; IVF, in vitro fertilization; OPU, ovum pick‐up.

^a^ Median (IQR); *n* (%).

^b^ Mann‐Whitney *U* test.

^c^ Chi‐squared test or Fisher's exact test.

^d^ Number excluding cycles with unknown pregnancy outcome.

^e^ Number excluding patients with unknown pregnancy outcome.

## 4 DISCUSSION

For patients with severely impaired sperm quality, ICSI is undoubtedly the treatment of choice to achieve pregnancy. In general, patients with very low quality and quantity of sperm in the ejaculate have poorer results with ICSI. Several studies support the use of testicular rather than ejaculated spermatozoa for ICSI in couples with virtual azoospermia or cryptozoospermia.^4^, ^5^, ^6^

It is known that the use of motile sperm for ICSI is an important predictor of ART results in terms of fertilization rate, pregnancy rate, and delivery rate, both in ejaculated sperm as well as in testicular sperm. The degree of sperm maturity has also an important impact on the outcome of ICSI. Esteves et al. in a systematic review and meta‐analysis reported when comparing sperm DNA fragmentation (SDF) levels between testicular and ejaculated sperm, testicular sperm had lower SDF.^7^ They concluded fertilization rates were not different between sperm sources, however, clinical pregnancy and live birth rates were higher for testicular than for ejaculated sperm, whereas miscarriage rates were reduced with testicular sperm.^7^ Some authors have also reported that elevated SDF in subjects with obstructive azoospermia resulted in poorer fertilization and pregnancy rates.^8^, ^9^ On the other hand, Stalf et al.^10^ reported that irrespective of sperm origin, the fertilization potential of injected spermatozoa was influenced by motility. Nicopoullos et al. also concluded from a meta‐analysis of published data in obstructive and NOA, that the etiology of azoospermia and cryopreservation of surgically harvested sperm affects ICSI outcomes, but the origin of the sperm does not affect the outcome of ART.^11^ Ku et al. in another meta‐analysis reported that no significant difference in miscarriage between testicular sperm and ejaculated sperm subjects, yet, take‐home babies per embryo transfer using testicular sperm was higher compared to ejaculated sperm.^12^ On the other hand, Abhyankar et al. reported in another meta‐analysis, there were no differences in pregnancy rate or fertilization rates between testicular and ejaculated sperm groups. They concluded that the existing literature does not support a recommendation in cryptozoospermia to use testicular sperm in preference over ejaculated sperm for ICSI.^13^ Ben‐Ami et al. reported that TESE is only justified in patients with cryptozoospermia who failed to conceive by ICSI using ejaculated spermatozoa, as it offers a higher pregnancy rate.^14^ Recently, the guidelines presented by Esteves et al. state that the evidence supporting the recommendation has increased in recent years in SDF testing, but is still of moderate to low quality.^15^ The impact of SDF on ART still remains unclear. Thus, previous studies have not clearly indicated whether cryptozoospermic subjects should use testicular sperm or ejaculated sperm for ICSI and this comparison is still controversial. In obstructive azoospermia, epididymal sperm have been reported to have higher SDF than testicular sperm.^6^ Although we have not evaluated SDF, we believe that SDF does not significantly affect ART outcomes because ICSI using epididymal sperm is associated with high clinical pregnancy and delivery rates.^16^

Micro‐TESE has been employed widely used as sperm retrieval does not require precise microsurgical skills. However, there have been reports of postoperative lower testosterone levels with TESE.^17^, ^18^ In addition, TESE requires tissue mincing and the cryopreservation process is complicated, leading to an increase in the laboratory workload for embryologists. If the results of ART are the same in ICSI using ejaculated sperm and sperm obtained by TESE, TESE should be avoided due to the aforementioned points. In the present study, although the cryptozoospermia group had fewer number of mature oocytes (4 vs. 8, *p* = 0.00148) and a significantly lower number of fertilized eggs (3 vs 6, *p* = 0.00112) than the TESE group, there was no difference in the normal fertilization rate (70.4% vs 72.1%). Similarly, the cryptozoospermia group had a lower clinical pregnancy rate (31.5% vs 43.6%, *p* = 0.01211) and live birth rate (20.5% vs 31.3%, *p* = 0.01308) per embryo transfer, but there was no significant difference in live birth per patient (73.8% vs 87.5%, *p* = 0.118). These ART results also reflect female factors such as low numbers of mature oocytes rather than sperm quality.

It is mandatory to have good laboratory procedures in place to allow sperm identification in semen, sperm processing, and undertaking the ICSI technique through employing highly trained embryologists. Our results showed that ART can be carried out in cryptozoospermic subjects without sperm retrieval surgery resulting in a live birth rate per case of 73.8%. This suggests that the poor ART results previously reported in cryptozoospermia, is not always due to sperm quality. If motile sperm are presented in ejaculates of cryptozoospermic subjects, invasive sperm retrieval surgery may be avoided.

### 4.1 Limitations, reasons for caution

The present study was retrospective and single institutional setting.

#### 4.1.1 Wider implications of the findings

Since the ART results with the use of ejaculated sperm from cryptozoospermic patients were comparable to the TESE results, traditionally poor ART results could not be due to sperm.

## 5 CONCLUSIONS

If cryptozoospertmic subjects present with motile sperm in their ejaculates, physically and endocrinologically invasive sperm retrieval surgery can be avoided.

## FUNDING INFORMATION

Each author has no COI with regard to this manuscript.

## CONFLICT OF INTEREST STATEMENT

The authors declare no conflict of interest.

## DISCLOSURES

*Human rights and informed consent statements*: All procedures completed were done in accordance with the ethical standards of the responsible committee on human experimentation (institutional and national), and with the Helsinki Declaration of 1964 and its later amendments. Informed consent was obtained from this patient for the purpose of inclusion in this study.

## ETHICS STATEMENT

The protocol for this research project, including its use of human subjects, was approved by a suitably constituted Ethics Committee (our approval number: 2020‐14, Date of approval by the Ethical Review Committee: 2020/09/30).
